# Förster Resonance Energy Transfer (FRET) as a Tool for Dissecting the Molecular Mechanisms for Maturation of the *Shigella* Type III Secretion Needle Tip Complex

**DOI:** 10.3390/ijms131115137

**Published:** 2012-11-16

**Authors:** Nicholas E. Dickenson, William D. Picking

**Affiliations:** Department of Microbiology and Molecular Genetics, Oklahoma State University, Stillwater, OK 74078, USA; E-Mail: william.picking@okstate.edu

**Keywords:** Förster resonance energy transfer (FRET), Type III secretion system, *Shigella flexneri*

## Abstract

Förster resonance energy transfer (FRET) provides a powerful tool for monitoring intermolecular interactions and a sensitive technique for studying Å-level protein conformational changes. One system that has particularly benefited from the sensitivity and diversity of FRET measurements is the maturation of the *Shigella* type III secretion apparatus (T3SA) needle tip complex. The *Shigella* T3SA delivers effector proteins into intestinal cells to promote bacterial invasion and spread. The T3SA is comprised of a basal body that spans the bacterial envelope and a needle with an exposed tip complex that matures in response to environmental stimuli. FRET measurements demonstrated bile salt binding by the nascent needle tip protein IpaD and also mapped resulting structural changes which led to the recruitment of the translocator IpaB. At the needle tip IpaB acts as a sensor for host cell contact but prior to secretion, it is stored as a heterodimeric complex with the chaperone IpgC. FRET analyses showed that chaperone binding to IpaB’s *N*-terminal domain causes a conformational change in the latter. These FRET analyses, with other biophysical methods, have been central to understanding T3SA maturation and will be highlighted, focusing on the details of the FRET measurements and the relevance to this particular system.

## 1. Introduction

Förster (non-radiative) resonance energy transfer (FRET) has provided a powerful tool that is readily applied to monitoring interactions between molecules as would occur upon binding of a ligand to a protein or the association of a protein to another protein. In the former situation, it can even be used to localize a ligand binding site relative to other known sites on the protein that binds it. FRET also represents an exceptionally sensitive technique for detecting changes in intramoleuclar distances and as a molecular ruler for determining the magnitude of protein structural changes. A model system that has been the focus of research by our group and which has benefited significantly from the use of FRET and other fluorescence spectroscopic methods is maturation of the *Shigella flexneri* type III secretion apparatus (T3SA) needle tip complex. In the review that follows, we spell out the unique inroads that FRET has allowed us to make in defining the molecular events that lead to T3SA needle tip maturation. While the FRET data presented here have served to complement other biophysical data in generating a model for the first steps of type III secretion induction, the story is one that would be incomplete without the mechanistic data this methodology provides.

The T3SA is used by *Shigella* to deliver effector proteins into human intestinal cells to promote bacterial entry as the first step in the onset of dysentery [1]. The T3SA is comprised of an intricate basal body that traverses the bacterial envelope and a needle with an exposed tip complex that matures in response to environmental stimuli [2]. FRET and fluorescence polarization have been used to demonstrate bile salt binding by the nascent needle tip protein invasion plasmid antigen D (IpaD) [3] as well as to identify and describe conformational changes that occur within IpaD following bile salt binding [4]. These events have been proposed to promote the recruitment of a second protein, IpaB, to the T3SA needle tip where it then senses contact with host cell membranes as a final step in secretion induction [5]. Prior to its secretion, IpaB is stored as an “inactive” heterodimer with its cognate chaperone. Through the use of FRET, we have revealed that chaperone binding greatly affects IpaB structure [6] and this, in turn, limits IpaB’s ability to oligomerize and to interact with phospholipid membranes. In this review dedicated to the use of FRET as a valuable analytical too, we will focus on the multiple contributions that FRET has provided in dissecting the discrete steps in maturation of the *Shigella* T3SA and mention novel uses of this technique by other groups also exploring type III secretion.

## 2. Use of Fluorescence in Exploring the Lives of Bacteria and Bacterial Pathogens

Fluorescence techniques have been instrumental in the study of biological systems on many scales ranging from protein-protein interactions to exploring tissue make up to examining the localization of molecules within complex organisms. Much of the success of fluorescence techniques is due to its inherent advantages. Fluorescence measurements are generally noninvasive and offer high temporal resolution, high specificity, polarization and spectroscopic capabilities, and low detection limits. The wide-spread use of fluorescence techniques has fueled the development of a vast library of fluorophores that span beyond the visible spectrum. Furthermore, the use of appropriate filter combinations has allowed the inclusion of multiple fluorescent probes in a single experiment. While many commercial probes are available directly bound to macromolecules such as antibodies or small/large receptor ligands, some are made with specific reactive moieties allowing them to be site-specifically coupled to molecules of the investigator’s choosing, providing great flexibility in their uses. For example, fluorescence has been widely used to explore the many functions of bacteria and bacterial pathogens. Image analysis using fluorescence microscopy has been invaluable in identifying bacterial dynamics [7], the fate of intracellular bacterial pathogens [8,9], biofilm structure [10], and the subcellular events occurring within bacteria [11,12]. Fluorescence techniques have also been widely used to identify and study bacterial virulence factors such as the type III secretion system (T3SS) expressed in many different pathogens including *Shigella flexneri* (the subject of this review).

### 2.1. Limitations of Traditional Fluorescence Measurements and Alternative Techniques

Despite the many advantages and widespread use of fluorescence measurements, they tend to suffer from a shared physical limitation. The maximal spatial resolution of conventional optical measurements is limited by the ability of a lens to focus light. This diffraction is well-characterized and depends on many variables, but limits the resolution to approximately half the wavelength of the excitation light (~250–300 nm for the visible spectrum) [13]. As many biological structures, interactions, and especially protein conformation/dynamics occur on much smaller scales, several other microscopy/spectroscopy approaches have been developed to address this issue. High-resolution microscopy techniques such as transmission electron microscopy (TEM) and scanning electron microscopy (SEM), for example, are capable of achieving resolution on the nanometer scale and have been extensively used for the study of membrane organization [14,15], macromolecular protein complex formation and architecture [16,17], and pathogen/host relationships [18] to name just a few. Unfortunately, while these techniques offer orders of magnitude improvement in spatial resolution over traditional optical fluorescence detection and have proven invaluable in many studies, sample preparation is extensive and the samples must be imaged under vacuum, thereby limiting its use in dynamic solution-based applications. More recently, there have been a number of “super resolution” optical techniques developed which also offer a substantial increase in resolution, but they generally require expensive custom instrumentation and in many cases still potentially lack the resolution necessary for probing specific protein-protein interactions and resulting Å-level conformational changes important in understanding the system in question [19,20].

One fluorescence method that maintains the many advantages of fluorescence measurements while providing superior spatial sensitivity is Förster resonance energy transfer. FRET is a technique that offers the benefits of traditional fluorescence while providing Å-level sensitivities to changes the distance between the fluorophores [13,21]. It has proven to be a powerful technique for studying many aspects of microbiology on a biologically relevant scale [22,23]. We have used many aspects and experimental variations of FRET to complement more traditional microscopy and spectroscopy techniques to dissect the mechanism of the maturation of the T3SS needle tip complex in *Shigella flexneri*[3,4,6]. Here, we will focus primarily on this work, beginning with a review of T3SSs followed by a discussion of FRET and its relevant applications.

## 3. Type III Secretion and Secretion Systems

Gram-negative bacteria have evolved to possess numerous systems for delivering proteins across their inner (cytoplasmic) and outer membranes [24]. The movement of proteins out of the cell, whether into the periplasm or into the extracellular milieu, is essential for bacterial survival. Secreted proteins are needed for cell wall biosynthesis, biogenesis/maintenance of the outer membrane, transport, modification of the bacterial environment (e.g., hydrolysis of macromolecules as food sources, detoxification or the release of toxins that enhance competitive robustness), establishment of microbial communities (e.g., attachment to biotic or abiotic surfaces and communication with other bacteria), the conjugal transfer of genetic material, and many other essential functions [25]. There has long been an appreciation of the importance of protein secretion and this is underscored by the fact that nearly a fifth of the proteins produced by bacteria are destined to reside at least partially outside of the cell [25]. Most of these proteins are secreted by the general secretory pathway (GSP) which entails transport into or across the cytoplasmic membrane via a secretion signal located at the proteins’ *N* terminus [25] and which is recognized by the Sec machinery [26]. Translocation across the cytoplasmic membrane is accompanied by cleavage of the Sec signal peptide by the type I signal peptidase. Because they possess a double membrane system, Gram negative bacteria often secrete proteins into the extracellular milieu using steps that go beyond the Sec-dependent pathway. These additional pathways show conservation between the Gram-negative bacteria which has allowed them to be classified based on the nanomachines that are used to carry them out. In many cases, Sec-dependent export of proteins into the periplasm is critical for the assembly of these secretion systems since they often require complex machinery that resides within the inner membrane, the outer membrane, and within the periplasm [25].

**Figure 1:**
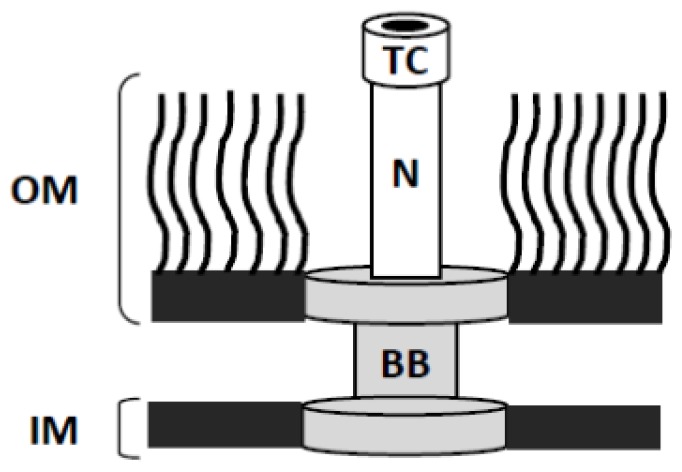
Cartoon representation of the type III secretion apparatus. The T3SA is comprised of three main components. The basal body (BB) spans both the inner (IM) and outer (OM) membranes of the bacterium anchoring the apparatus tightly to the pathogen. The T3SA needle (N) is comprised of a polymer of a single protein specific to the organism expressing the apparatus. The needle structure extends just beyond the O-antigen oligosaccharide portion of the OM lipopolysaccharides (LPS) anchored within the outer membrane and terminates with a pathogen-specific macromolecular tip complex (TC). Together, these components provide a unidirectional conduit that allows transfer of host altering effectors directly from the bacterial cytoplasm to the host cell.

One such example and a shared virulence factor for many Gram-negative pathogens is the T3SS [24]. Well studies paradigms for T3SSs include those used by the Enteropathogenic *Escherichia coli* (EPEC), *Salmonella*, *Yersinia*, and *Shigella*. While the illnesses caused by these pathogens vary dramatically, in all cases the T3SS is an essential virulence factor known to contribute directly to the onset and progression of infection. In each case, the T3SS provides a mechanism by which the bacteria communicate with targeted eukaryotic cells to subvert normal cellular processes for the benefit of the pathogen. Much of what is known of the ultrastructure of the T3SA stems from transmission electron microscopy of the systems encoded by *Shigella flexneri* and *Salmonella typhimurium*[27,28]. In these organisms (and *Yersinia*) the T3SA resembles a nano-syringe and needle (Figure 1) and represents the energized apparatus that is used by the T3SS to translocate effectors from the bacterial cytoplasm to the host cell cytoplasm. The T3SA is composed of 25 or more different protein types, the majority of which are dedicated to forming the basal body of the “needle complex” that spans the bacterial inner and outer membranes. Located at the cytoplasmic face of the T3SA are an energizing ATPase and regulatory proteins that act as substrate switches needed to allow coordinated assembly of the needle complex and provide the hierarchy of translocator/effector protein secretion [29]. The remaining “structural” components of the needle complex basal body consist of: (a) the inner membranes rings imbedded in the cytoplasmic membrane; (b) the periplasmic or inner rod that spans the periplasm; and (c) the outer membrane ring that connects the base with the extracellular portions of the T3SA [27,28].

Extending outward from the base, the T3SA possesses an external needle with an associated “tip complex” [16,30]. These are the structures exposed to the extracellular environment where they would be expected to have a major role in sensing the environmental signals that lead to type III secretion induction [5]. The T3SA needle tip complex was only recently discovered in the *Yersinia* and *Shigella* systems [31,32], however, it is likely that such “tip complexes” exist in all T3SAs. While the sensing and triggering mechanisms for induction of type III secretion are only now beginning to be studied, it is likely that contact between the T3SA “tip complex” and environmental signals is critical for this process. The progression of events that occur in secretion induction and the role of the tip complex and needle in this process will be discussed in more detail below, but it is clear that the first step in type III secretion induction is the recruitment of the “translocator” proteins to the tip of the needle, presumably in a stepwise manner as we have shown occurs in *Shigella*[5,33].

The translocator proteins (IpaB and IpaC) ultimately form the “translocon” pore within the target cell cytoplasmic membrane and it is through this pore that effector proteins are likely to gain access to the host interior [34]. While the mechanism of pore formation remains somewhat controversial, the size of the translocon pore has been determined for several systems and it is generally considered to have an inner diameter between 1.5 and 3.5 nm based on contact hemolysis assays done in the presence of different sized osmoprotectants [35]. In *Shigella*, completion of the translocon pore triggers full secretion of effector proteins from the bacterial cytoplasm [33], through the completed T3SA, and into the host cytoplasm. Once injected, the effector proteins subvert many of the normal host cell functions promoting uptake of the pathogen and evasion of the host’s immune defenses [24].

### 3.1. Shigella as a Model for Dissecting the Structure and Function of the T3SA Needle Tip Complex

*Shigella* species are the etiologic agents of shigellosis, a potentially life-threatening bacillary dysentery that is restricted in host range to humans and other higher primates [1]. While shigellosis is recognized as a disease of the developing world, it is greatly underreported in industrialized regions of the world. Following ingestion of contaminated water, *Shigella* crosses the colonic epithelium, kills macrophages, and actively enters intestinal epithelial cells by inducing macropinocytosis [36]. *Shigella*’s ability to invade epithelial cells is entirely dependent upon its T3SS. The *Shigella* T3SA is structurally similar to T3SAs from many other pathogens and is encoded by the *mxi/spa* loci with major secreted proteins including the invasion plasmid antigens (Ipa proteins) which have both translocator and effector functions [24]. The external needle of the *Shigella* T3SA is composed of a homopolymer of MxiH and is about 45 nm in length with a 7-nm outer diameter and a central channel with a diameter of 2.5 nm [37]. It was recently found that prior to secretion induction, IpaD stably resides at the tip of the *S. flexneri* T3SA needle, suggesting its recruitment directly follows termination of MxiH needle synthesis [31]. Interestingly, at this point other Ipa proteins were not found to be abundantly exposed at the needle tip of these bacteria, however, the translocators IpaB and IpaC are detected by immunoblot in whole cell preparations and IpaB was detected in artificially long needles sheared from the *Shigella* surface [31]. These data support a discrete stepwise model of *Shigella* T3SA tip complex assembly in which all of the needle tip complex components are expressed and available in the bacterial cytoplasm, but await an environmental signal to promote translocation through the needle and inclusion into the maturing needle tip complex.

### 3.2. High-Resolution Structures of the External Portions of the T3SA

The most prominent extracellular component of the T3SA is a needle that is assembled from multiple copies of a single small low-pI protein (MxiH in *Shigella*) [38]. Assembly of the needle is believed to occur at the tip of the growing apparatus as has been shown for the analogous flagellar hook and FliC filament structures [39]. This requires that needle monomers pass through the T3SA central channel to polymerize at the growing distal end of the needle. Type III secretion fails to occur in the absence of an assembled needle and mutations in the needle protein that block its ability to polymerize result in secretion of soluble needle monomers without a switch to subsequently exported substrates, including needle tip complex proteins and translocator proteins [38]. Because of the self-polymerizing nature of needle proteins from diverse pathogens [40], structure determination was initially difficult, however, complementary methods in X-ray crystallography and NMR spectroscopy allowed determination of needle monomer structures for MxiH (*Shigella*), BsaL (*B. pseudomallei*) and PrgI (*Salmonella*) [41–43]. The first model of an assembled T3SS needle was generated by docking the crystal structure of MxiH onto a 16-Å density map of needles sheared from the *Shigella* surface using three-dimensional electron microscopy reconstruction, providing an atomic picture of the complex needle structure [44].

Scanning transmission electron microscopy (STEM) analysis subsequently provided the first evidence of a stable structure at the tip of a T3SA needle with LcrV from *Y. enterocolitica* atop isolated needles [32]. Shortly after, it was shown that IpaD also forms a tip complex for the *S. flexneri* T3SA when the organism is grown to early log phase in trypticase soy broth (TSB) [31]. With no other proteins detected as part of the *Yersinia* or *Shigella* tip complexes in these pioneering studies and its position as the outermost point of the T3SA, it was proposed that the identified needle tip complex is directly involved in sensing environmental signals and therefore has a role in controlling the secretion status of the T3SS. There is currently much more direct evidence regarding IpaD as a regulatory component of type III secretion than there is for LcrV, perhaps due to the existence of more stringent control of secretion steps in the well-characterized *Yersinia* system [45]. These initial findings fueled a series of studies directed toward understanding the specific role(s) of IpaD as a T3SA tip complex protein, resulting in strong evidence for an IpaD role in sensing environmental signals. Specifically, these hypotheses stem from recent findings that it specifically binds bile salts to stimulate the recruitment of IpaB to the *Shigella* T3SA needle tip where it forms a stable structure because of its ability to associate with IpaD [5], which is driven at least in part by significant conformational changes in the structure of IpaD following DOC binding [4,46]. Unfortunately, it is still unclear how this action of IpaD specifically relates to that of other T3SA needle tip complex proteins, however, this may change with the ever growing use of novel techniques and as interest in the action of IpaD homologues increases.

Maturation of the needle tip complex is *Shigella* occurs when the translocator proteins (IpaB and IpaC) sequentially join IpaD at the needle tip. It was shown that bile salts promote recruitment of IpaB into the T3SA needle tip complex in *Shigella* and this appears to be mediated by the bile salt’s association with the needle tip protein, IpaD [3,5]. This implicates IpaB as a component of the maturing tip complex as it prepares for host cell membrane contact and its transitioning into a component of the translocon pore that will form in the host cell membrane. Indeed, the addition of liposomes containing sphingomyelin and cholesterol are able to induce recruitment of the second translocon pore component, IpaC, to the needle T3SA needle tip concomitant with full induction of type III secretion [33]. It is likely that a similar event occurs when the *Yersinia enterocolitica* translocators YopB and YopD are found to insert into erythrocyte membranes when the two are mixed [47]. While it thus seems likely that the T3SS translocators from many organisms insert into membranes upon contact to form pore-like structures, the failure to observe translocator proteins as part of the T3SA needle tip complex for other organisms suggests that the *Shigella* T3SS may be unique in that it is the most easily manipulated in the absence of a host cell (*i.e*., *in vitro*).

Fluorescence methods have had a prominent role in developing our current level of understanding of the mechanisms for temporal assembly of the *Shigella* T3SA needle tip complex. Most notably, immunofluorescence microscopy, fluorescence polarization spectroscopy, and FRET have revealed key events, interactions, and protein movements that occur during tip complex maturation. The remainder of this short review will describe how FRET has been used in this system to provide insight into the molecular events that immediately precede *Shigella* invasion of human intestinal epithelial cells.

## 4. Förster Resonance Energy Transfer

**Figure 2:**
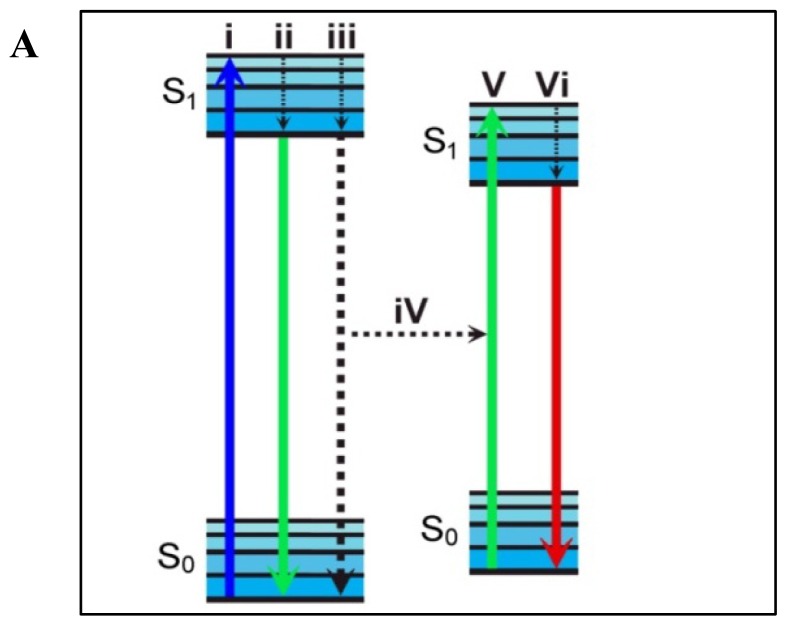

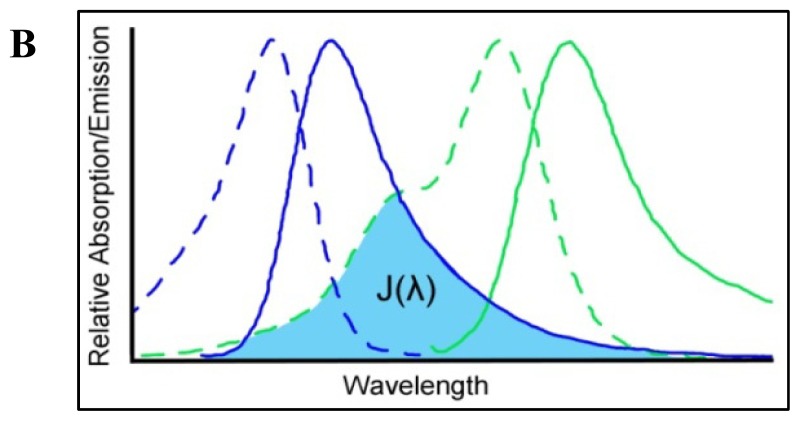
Panel **A** shows the coupled transitions that occur between an excited state FRET donor and ground state FRET acceptor within the context of a Jablonski diagram. Absorption of radiation of the correct energy promotes the excitation of the donor fluorophore to an electronic excited state (**i**) which quickly decays through vibrational relaxation as illustrated with the short dashed arrows. The excited state fluorophore can now return to ground state through several pathways including emission of a photon (**ii**). If an appropriate FRET acceptor is located near the excited state donor, it can undergo a non-radiative relaxation event (**iii**) in which the associated energy of the system is non-radiatively transferred to the ground state acceptor (**iv**) resulting in its elevation to an appropriate electronic state (**v**). The excited state acceptor can then eventually return to ground state through the same options as for the donor, including emission of a photon (**vi**). For clarity, several energy transitions not directly pertaining to FRET, such as rotational energy levels and intersystem crossing were excluded from the diagram. Panel **B** shows the excitation/emission spectra for a hypothetical FRET pair. The efficiency of energy transfer events outlined in panel **A** are dependent on several factors with potentially the most easily controlled (through choice of fluorophores) being J(ë) or the spectral overlap integral of the donor emission (solid blue) and acceptor excitation (dashed green).

Förster Resonance Energy Transfer is a technique that takes advantage of the non-radiative decay of an electronic excited state donor fluorophore to a nearby ground state acceptor probe (Figure 2A). Several extensive reviews detail the mechanisms driving the energy transfer [21,48] so here we will provide a simple overview before discussing specific examples. Many factors play a role in the efficiency of energy transfer from donor to acceptor and an equation relating these factors can be written as:

(1) $$R_{\text{o}}={\left[9.78\times 10^{3}\times K^{2}\times {\text{n}}^{-4}\times QY_{\text{D}}\times \text{J}(\lambda )\right]}^{1/6}Å$$

where *R*_o_ represents the distance between the fluorophores at which 50% energy transfer occurs. *K*^2^ is a dipole orientation factor which describes the relative orientation of the emission dipole of the donor fluorophore and the absorption dipole of the acceptor fluorophore. Since *K*^2^ is difficult to measure for fluorophores in solution, it is often approximated to be 2/3 for a freely rotating FRET pair. *QY*_D_ is the fluorescence quantum yield of the donor in the absence of an acceptor and J(ë) represents the integral of the spectral overlap between the donor emission and the acceptor absorption (Figure 2B). The determined *R*_o_ value can then be related to the energy transfer efficiency in the system by:

(2) $$E={R_{\text{o}}}^{6}/({R_{\text{o}}}^{6}+r^{6})$$

where again, *R*_o_ is the Förster distance constant for the chosen FRET pair and *r* is the distance separating them in Å. This relationship not only allows for calculation of the distance between the FRET fluorophores, but more importantly it describes the dependence of energy transfer on the distance between the probes as being to the inverse sixth power. This becomes very important as it means that Å-level changes in distance result in measureable changes in energy transfer efficiency, making FRET a powerful tool sensitive to conformational changes and interactions.

### 4.1. Measuring FRET

**Figure 3:**
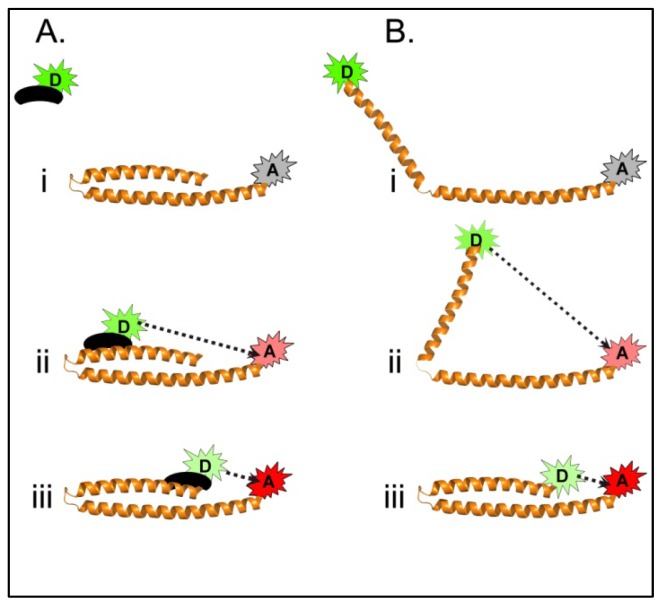
Schematic of hypothetical FRET systems probing intermolecular (**A**) and intramolecular (**B**) energy transfer. (**A**) In this intermolecular example, an acceptor fluorophore is bound to a protein of interest and a donor fluorophore is attached to a ligand which could represent a small molecule, peptide, or another protein. When unbound (**Ai**), the ligand-associated donor is separated by too much distance to allow efficient energy transfer to occur. Upon binding of the ligand to the protein, the binding event can be monitored by an increase in energy transfer efficiency (seen by the dashed line in **Aii** and **Aiii**). Depending on the complexity of the protein structure, the measured energy transfer efficiencies and calculated distances between the probes can be used to help identify the relative location of the ligand binding site (**Aii** and **Aiii**). (**B**) Measurement of intramolecular FRET can be used to gain insight into protein conformations and conformational changes following a specific event such as a molecular interaction. In this example, a protein conformational change results in the intramolecular FRET pair moving from a distance too great for energy transfer to be reliably observed (**Bi**), to an intermediate distance with detectable, but minimal, energy transfer (**Bii**), to a final conformation resulting in the two probes being within close proximity to each other and which gives rise to a maximal observed energy transfer efficiency (**Biii**).

There are several ways to experimentally measure energy transfer efficiencies while the choice of technique is often driven by the availability of equipment and the specifics of the system investigated. Potentially the most desirable method for calculating energy transfer involves the measurement of the fluorescent lifetimes of the donor fluorophore both in the presence and absence of the acceptor probe. The relationship is defined as:

(3) $$E=\left[1-\left(\frac{T_{\text{DA}}}{T_{\text{D}}}\right)\right]$$

where *E* again represents the energy transfer efficiency in the system, *T*_DA_ is the excited state lifetime of the donor fluorophore in the presence of the acceptor and *T*_D_ is the excited state lifetime of the donor in the absence of the acceptor. Because an additional relaxation pathway is present for the excited state donor when the FRET acceptor is present (Figure 2A), a decrease in excited state lifetime inversely proportional to the sixth power of the distance between the probes is expected. This allows for the calculation of not only the energy transfer efficiency (Equation (3)), but also the distance between the FRET probes in the system (Equation (2), Figure 3). The concentration-independent fluorescence lifetimes are measured by pulsed or phase resolved techniques that are capable of identifying multiple donor lifetimes resulting from discrete donor microenvironments and numerous non-radiative pathways, providing a reliable measure of energy transfer efficiencies. Despite the many advantages of this technique, it has had very limited use in the study of human pathogens likely because it requires sensitive specialized equipment and that the sample be exposed to either multiple pulses or longer periods of high intensity continuous wave radiation which are not compatible with all sample conditions, however, advances in instrumentation have recently allowed its expansion into more sensitive systems. For example, a recent study by Kopp *et al.* demonstrated the sensitivity and utility of coupling fluorescence lifetime imaging microscopy (FLIM) with acceptor photobleaching techniques to characterize protein interactions at the site of *Neisseria gonorrhoeae* interaction with host cells [49]. In that work, the authors measured donor fluorescence lifetimes prior to and following selective photobleaching of the acceptor to precisely calculate energy transfer efficiencies. This technique eliminates the need to prepare multiple sample conditions and provides concentration independent FRET measurements. This recent demonstration will hopefully open doors for the further use of FRET FLIM in studying bacterial pathogens and pathogen/host interactions.

Another method used commonly for its compatibility with microscopy and its ability to measure FRET efficiencies without the need to prepare a separate “donor only” sample is donor recovery post-acceptor photobleaching. A fluorescence microscope is used to quantify the donor fluorescence intensity in the sample both before and after selectively photobleaching the acceptor fluorophore in a defined region. The resulting loss of a non-radiative pathway results in a measured increase in donor fluorescence in this region. This difference in donor fluorescence is quantified and normalized against the donor signal in the presence of active acceptor in order to calculate the energy transfer efficiency between donor and acceptor dyes prior to photobleaching the acceptor. This technique is popular for its ability to measure FRET efficiencies in specific regions of a sample as well as to directly visualize both donor and acceptor fluorophore distributions within the sample. One excellent example of its use in studying bacterial pathogenesis was by Isberg and co-workers in a study characterizing the differential effects of two *Yersinia* T3SS effector proteins on Rac1 activation/suppression and membrane localization [50]. Here, they were able to not only probe cellular Rac1 localization following *Yersinia* interaction with host cells, but found that the pathogen specifically suppressed Rac1 activation in the cytoplasm while Rac1 remained active in the nucleus, suggesting a novel method of control over host cell function by spatially isolating Rac1 activity [50]. While this technique offers many advantages such as those demonstrated above, it suffers from limitations surrounding sample preparation. For example, it requires that the sample be immobilized to allow acceptor photobleaching in a defined region without the interference that would be caused by fluorescent probes that are able to diffuse in or out of the photobleached region. This is most conveniently achieved by chemical fixation.

A third and one of the most common methods for quantifying solution-based FRET also takes advantage of the difference in donor fluorescence levels when in the presence or absence of an appropriate acceptor and can be quantified by the following equation:

(4) $$E=\left[1-\left(\frac{F_{\text{DA}}}{F_{\text{D}}}\right)\right]$$

where *E* is again the quantified energy transfer efficiency, *F*_DA_ is the FRET donor fluorescence in the presence of acceptor and *F*_D_ is the donor fluorescence in the absence of acceptor. This technique carries the advantages of requiring only a simple fluorometer and very small amounts of sample to measure FRET under several conditions including in solution, but like all steady state FRET measurements monitoring donor intensity, it requires that the concentration of the system components be known precisely. This method has been used extensively in the study of the *Shigella* T3SS to monitor and characterize interactions and protein conformational changes with Å-level sensitivity [3,4,6].

All of the approaches discussed above take advantage of the difference in fluorescence properties (intensity and lifetime) of the FRET donor depending on the level of energy transfer between the probes. Alternatively, energy transfer can be quantified by measuring FRET acceptor fluorescence since it is expected to increase proportionally to the observed decrease in donor fluorescence or *vice versa*. This technique, however, suffers from several technical drawbacks such as non-FRET based fluorescence resulting from spectral overlap of the excitation source and the excitation profile of the acceptor. For these reasons it is not commonly used. This and many other factors that must be taken into account when designing a FRET experiment are highlighted and discussed below.

### 4.2. Consideration of FRET Limitations

#### 4.2.1. Donor and Acceptor Labeling

An important consideration in any FRET experiment, especially in the case of biological applications involving proteins, is the choice of the FRET donor-acceptor pair. Because measured energy transfer efficiency is related to the distance between the FRET pair (*r*) to the inverse sixth power (Equation (2)), it is most sensitive to changes when r is near the *R*_o_ value. If the investigator has some insight to this distance *a priori*, a donor acceptor pair with an appropriate *R*_o_ value should be chosen. If not, an estimate based on the dimensions of the system under study is usually adequate. Once chosen, it is necessary to incorporate the fluorophores into the system. This can be achieved by an ever expanding number of methods. Many biologically important molecules such as signaling and structural components and phospholipids are available commercially with covalently bound fluorescent tags. In the case of less common small molecules, fluorescent versions can be synthesized as will be discussed below for the case of the bile salt deoxycholate. When considering more complex molecules such as proteins, on the other hand, many things have to be taken into consideration. A vast library of reactive fluorophores are commercially available and can be used to specifically label targeted sites within the protein structure. Of the most common are primary amine- and sulfhydryl-reactive probes that primarily target lysine and cysteine side chains, respectively.

While the typical higher abundance of lysine residues in proteins and the presence of a primary amine at the *N*-terminus of all proteins generally allows for a high labeling efficiency, it can also result in multiple fluorophores per protein and a heterogeneous distribution of labeled sites. For proteins that contain only one cysteine residue, a sulfhydryl-reactive probe offers site-specific labeling and there is always the flexibility of mutating a protein sequence to include a single cysteine at a desired location. This is not without its own set of challenges, however, as altering the protein sequence means that it is not only necessary to ensure that addition of the fluorophore does not negatively impact the protein’s characteristics (*i.e*., secondary structure, tertiary structure, ability to bind ligand, *etc*.), but also that the mutation does not alter protein structure or function. Targeting exposed loops or regions outside of defined secondary structure components such as á-helices and â-sheets generally reduces the impact of both the labeling and the mutation on the protein’s properties. As with any alteration of protein primary structure, however, the properties of the substituted residues also need to be considered and designing several mutants for characterization and analysis is often a sound approach.

In the case of intermolecular FRET, optimization of donor and acceptor labeling can be done independently for the two interacting molecules. In contrast, intramolecular FRET requires that a separate donor and acceptor fluorophore is included into the same molecule (often a protein). This can be challenging because it is imperative that the labeling sites for each respective probe be well-defined and not cross-reactive. Many creative options for this have been devised to allow this. Some examples include the use of recombinant fluorescent protein fusions, the engineering of differential fluorophore-specific recognition sites, and the use of a mixture of natural intrinsic fluorophores with covalently bound extrinsic fluorescent probes. In this review, we will discuss several specific examples used to monitor intramolecular FRET in T3SS proteins; however, it is important to note that they only represent a fraction of the creative techniques that can be applied to meet the challenge of multi-probe labeling for FRET analysis.

#### 4.2.2. Technical Considerations

FRET is a powerful tool that has been used extensively to study biological systems and with the numerous techniques available for quantifying energy transfer, it has become a standard tool in many scientists’ toolboxes. When designing a FRET experiment, however, several factors such as the physical state of the system, the spectral properties of the donor and acceptor, limitations of available instrumentation, and the nature of the question being asked must be carefully considered. For example, when using steady state donor fluorescence levels in the presence and absence of acceptor to calculate FRET efficiencies (Equation (4)) it is imperative to ensure that the protein concentrations and donor labeling efficiencies in the samples containing only the donor label are identical to those containing donor and acceptor. It is also important to appropriately characterize the properties of the sample prior to initiating FRET measurement. Situations such as unforeseen oligomerization of proteins, the binding of multiple acceptor-labeled ligands to one donor labeled protein, or an inefficient labeling of a protein (especially with an acceptor probe) can result in difficulties interpreting results. For these reasons, changes in FRET efficiencies (distances) are often weighed more heavily than the absolute distances calculated from a rearrangement of Equation (2). Nevertheless, proper controls, a complete characterization of the system, and the use of complementary techniques can make FRET a valuable tool for understanding Å-level interactions and conformational changes that remain inaccessible by many other traditional techniques. The following provides a summary of a series of FRET experiments used by our group to provide structural and mechanistic insight into the stepwise maturation of the *Shigella* T3SA.

### 4.3. Using FRET to Dissect Shigella T3SA Maturation

#### 4.3.1. Using FRET to Identify Intermolecular Interactions That Occur During Maturation of the *Shigella* T3SA Needle Tip Complex

The bile salt deoxycholate (DOC) plays an important role in maturation of the T3SA and ultimately the virulence of *Shigella*. The addition of deoxycholate to *Shigella* cultures prior to incubation with cultured epithelial cells significantly increases the invasiveness of the organism as measured by a standard gentamycin protection assay. Both fluorescence and electron microscopy studies showed that exposure to DOC recruited the first T3SS translocator protein IpaB to the needle tip [5]. These results, together with the high physiological levels of DOC in the small intestine suggest that DOC induced IpaB recruitment is an important step in the “priming” of the *Shigella* T3SA following ingestion, however, the mechanism through which DOC acts remained unclear. To approach this problem, we synthesized a fluorescein labeled derivative of DOC (FITC-DOC) which could be used to investigate the IpaD-DOC interaction. Fluorescence polarization experiments demonstrated that FITC-DOC strongly and specifically binds to IpaD [3]. To confirm this interaction and provide a first step in determining the DOC binding site on IpaD, intermolecular FRET between a thiol-reactive coumarin maleimide covalently bound to the single native cysteine near the end of the IpaD central coiled-coil and the fluorescein-DOC was carried out [3]. Energy transfer efficiencies were calculated by comparing the coumarin donor fluorescence intensities following titration of unlabeled DOC to those of the labeled acceptor. The maximal energy transfer efficiency was found to be 78% and the *R*_o_ value determined to be 52 Å. These results confirmed the interaction between DOC and IpaD and estimated the IpaD/DOC binding site to be within approximately 42 Å of the donor-labeled cysteine at position 322. In good agreement with the FRET results, computational ligand docking simulations predicted that DOC binds in the cleft of IpaD’s central coiled-coil at approximately the center of the molecule. While the precise DOC binding pocket would later be modified based on co-crystallization studies [46], FRET provide a relatively accurate first approximation of the DOC binding site, providing a means for site-directed mutagenesis studies investigating the impact of local residues on the *Shigella* virulence.

#### 4.3.2. Identifying Ligand Binding Effects on Protein Structure—the Interesting Case of IpaD

**Figure 4:**
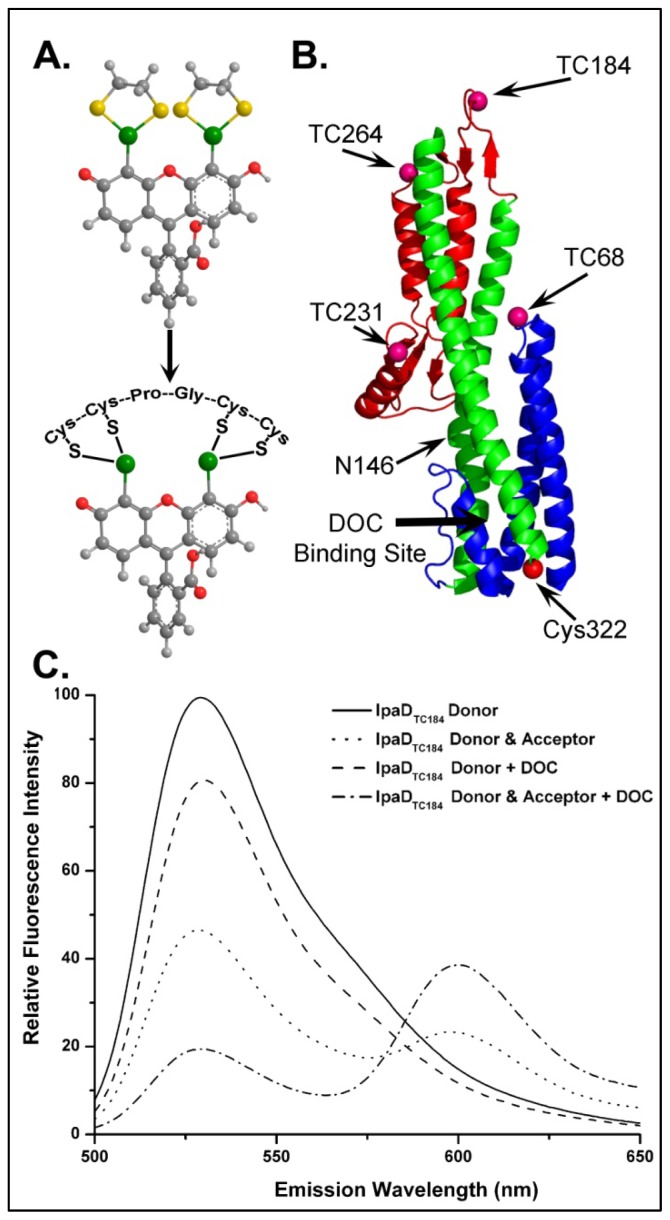
Location of tetracysteine (TC) FlAsH binding sites engineered into IpaD and the fluorescence emission spectra used to quantify FRET from FlAsH on TC184 to Alexa 568 on Cys322. FlAsH is a fluorescein-based fluorophore that specifically coordinates to the TC sequence Cys-Cys-Pro-Gly-Cys-Cys, resulting in a dramatic increase in quantum yield [51] (**A**). The residues at which TC pockets were introduced into IpaD are indicated on the ribbon structure (**B**). Panel **C** shows the representative fluorescence spectra used to determine the effect of DOC on energy transfer efficiencies between FlAsH and the acceptor (Alexa 568) at the native Cys in the IpaD TC184 mutant. As described in the text, energy transfer efficiencies were quantified by measuring the change in donor fluorescence emission intensity in the presence and absence of an acceptor. The spectra in panel **C** not only show that the donor emission is decreased in the presence of the FRET acceptor, but that the extent of the change is dependent on DOC, allowing a systematic measure of the effect of DOC binding on IpaD structure. The key for identification of each spectrum is given in the inset. Figure adapted from [4].

Now that we had clearly identified not only the importance of DOC on *Shigella* virulence phenotype, but also identified and characterized the IpaD/DOC interaction, we wanted to investigate the mechanistic importance of this event on the maturation of the *Shigella* T3SA. IpaD had already been speculated to reside atop the immature T3SA and act as a “plug” controlling the secretion of translocators and ultimately the late effectors [31], however, the signals for activating secretion remained unknown. We hypothesized that DOC interaction with IpaD results in a conformational change within the protein to promote the second step of T3SA maturation (IpaB recruitment to the tip complex). To test this, we once again turned to FRET measurements to identify and characterize conformational changes in IpaD following DOC interaction [4]. Specific locations within IpaD were labeled in order to investigate conformational changes within the protein via intramolecular FRET. The native cysteine at position 322 was targeted to covalently link an Alexa 568 fluorophore as a FRET acceptor and multiple locations for the placement of a FlAsH donor fluorophore were engineered into the primary sequence of the protein. FlAsH is a fluorescein-based biarsenical fluorophore that strongly and specifically coordinates to the tetracysteine motif Cys-Cys-Pro-Gly-Cys-Cys (Figure 4A) which was engineered into several regions of IpaD (Figure 4B). Biophysical characterization of the recombinant mutant and phenotype analysis of *Shigella* expressing the FlAsH-binding IpaD ensured that the mutants to be characterized *in vitro* by FRET were not negatively impacted by the inclusion of the FlAsH binding pockets or of the coordinated fluorophore. Four FlAsH mutants were identified as fully functional and each was labeled with the FlAsH donor fluorophore to near 100% efficiency (Figure 4B). A portion of the FlAsH-labeled IpaD was then treated with the thiol-reactive Alexa 568 acceptor, providing a FRET acceptor at the native cysteine (Cys322) located at the bottom of the coiled-coil (Figure 4B).

Energy transfer efficiencies were calculated for each donor/acceptor combination in the presence and absence of 1 mM DOC by quantifying the decrease in donor fluorescence emission in the presence of acceptor compared to an equal concentration of the mutant labeled only with the FlAsH donor probe (Figure 4C) [4]. The results identified global conformational changes in the IpaD protein structure with the most pronounced being an 11 Å decrease in the distance between the FlAsH donor at location TC184 near the top of the coiled-coil and the acceptor at the native cysteine located at the bottom of the structure (Figure 4B). Comparison of ^1^H-^15^N TROSY-HSQC NMR spectra of IpaD with titrated amounts of DOC also identified many residues throughout the protein with significant chemical shift perturbations following DOC exposure. In good agreement with the results from the intramolecular FRET measurements, many of the chemical shift perturbations were localized to the central coiled-coil, further suggesting that it represents the region of the protein most greatly affected by DOC binding even though it is well-outside the predicted DOC binding site.

Further analysis of the previously published IpaD crystal structure identified a Pi-bulge in helix alpha-3 (residues 146–149) near the bottom of the protein structure. This discontinuity in helical character could be responsible for the flexibility seen in the protein core following DOC exposure. Several mutations were made in this region in an attempt to stabilize the helix. Phenotype analysis of these mutants found the N146Q mutation to be of particular interest as it resulted in the stable recruitment of IpaB to the *Shigella* surface without the need for DOC exposure, suggesting that the mutation may mimic the DOC-bound state [4]. The N146Q mutation was incorporated into all of the previously generated IpaD FlAsH constructs and the intramolecular FRET measurements were repeated for the mutants. As with the wild-type IpaD, all of the N146Q mutants exhibited significant conformational changes within IpaD following DOC exposure, however, while most of the regions showed no effect from the N146Q mutation, the change in distance between the FlAsH donor at TC184 and the acceptor at Cys322 was reduced by greater than 5 Å. Together with ^1^H-^15^N TROSY-HSQC NMR measurements, these FRET results suggested that the IpaD N146Q mutation reduced the inherent flexibility of the Pi-bulge in á3, reducing the structural effects of DOC binding to IpaD. The use of intramolecular FRET in these experiments was critical for laying the foundation for these conclusions.

**Figure 5:**
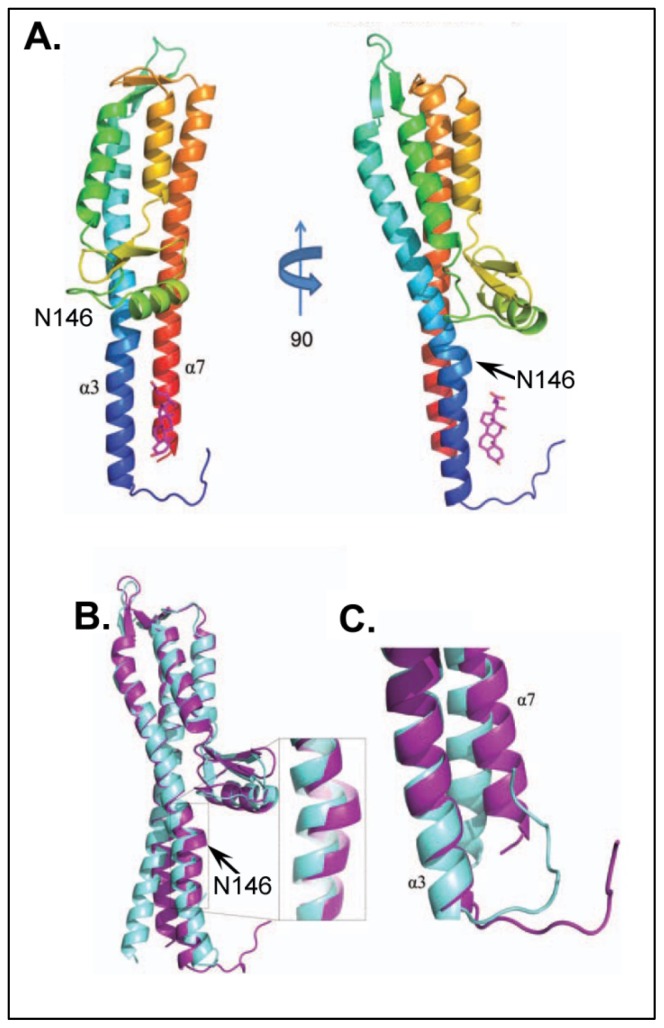
Panel **A** shows the 1.90 Å-resolution co-crystal structure of DOC bound to IpaD^122–319^ from *Shigella flexneri*. The right side is a 90° rotational view of the solved structure with IpaD in cartoon ribbon format and DOC located near the bottom of the molecule in the cleft of helices á-3 and á-7. Panel **B** and **C** show the superimposition of free IpaD^15–332^ (aquamarine) and DOC-bound IpaD^122–319^ (purple) structures (DOC removed for clarity) identifying several regions of IpaD affected by DOC binding, including the accentuation of the “kink” in helix á-3 (**B**) and the apparent torsion of the *C*-terminal region of helix á-7 (**C**) [46] both consistent with the conformational changes predicted by the previous FRET measurements [4]. Figure adapted from [46].

Continuing these studies, a 1.9 Å resolution co-crystal structure for IpaD with DOC was solved (Figure 5A) [46], which not only confirmed many of the conclusions regarding the interaction between IpaD and DOC, but also provided further insight into the potential mechanism of *Shigella* T3SA maturation. Although the identified binding location of the DOC was slightly lower on the IpaD structure than was predicted computationally (see above), the distance of the DOC from the location of the FRET donor-labeled Cys322 is in good agreement with the solved structure. Furthermore, a comparison of the crystal structures of the apo- and DOC bound IpaD confirmed a significant conformational change in the protein structure, primarily in the central coiled-coil and the region of the identified Pi-bulge (Figure 5B,C), as previously predicted by the intramolecular FRET and NMR results [4]. Taken together, the results at this stage not only identified and characterized the interaction between IpaD and DOC, but also allowed us to begin exploring the mechanism through which DOC elicits IpaB to the maturing needle tip of *Shigella*. Several more studies including both inter- and intra-molecular FRET analysis are currently underway to further characterize the dynamic relationship between IpaD, DOC, and IpaB.

#### 4.3.3. Monitoring the Structural States of the *Shigella* Translocator IpaB with an Intrinsic FRET Donor

The use of FRET to complement more traditional biochemical techniques has been instrumental in expanding our understanding of *Shigella* T3SS maturation from the role of IpaD to recruitment and involvement of the first hydrophobic translocator IpaB. Although the reasons are not entirely clear, IpaB exists in the bacterial cytoplasm only as a complex with its cognate chaperone IpgC and while the extent of the importance of this relationship remains unclear, it is known that the interaction stabilizes the hydrophobic character of IpaB, prevents its interaction with the pathogen’s cytoplasmic membrane, and inhibits premature interaction between itself and other T3SA components that are also stored in the cytoplasm [52]. The chaperone is removed by the T3SA ATPase prior to translocation through the needle, allowing IpaB to not only be secreted, but also to dock at the maturing T3SA needle tip where it is then responsible for sensing contact with eukaryotic host cell membranes and membrane components. While the *Shigella* system has allowed us to identify and follow these steps *in vivo*, the large size and hydrophobic nature of IpaB have made detailed *in vitro* studies involving the full length protein challenging. Recombinant expression of IpaB requires co-expression with IpgC followed by purification of the stable complex. Purified IpaB can then be isolated by incubation with a mild non-ionic detergent to remove the chaperone and stabilize the protein [53]. Unfortunately, the protein only remains stable in the presence of detergent, preventing *in vitro* characterization of many important interactions. To improve our chances for working with IpaB *in vitro*, limited proteolytic digestion was used to identify soluble domains [54]. These were initially used for structural analysis, however, the identified domains have also proven useful in studying specific protein-protein interactions involving IpaB.

These proteolytic digestion assays recently produced a large protease-resistant fragment of IpaB (IpaB^74–224^) for which a 2.1 Å crystal structure was solved [54], providing a platform for the creation of larger soluble *N*-terminal fragments of the protein (IpaB^1–226^ and IpaB^28–226^). These soluble fragments allowed us to begin a systematic dissection of the structural state of IpaB as it passes from the *Shigella* cytoplasm to the extracellular milieu beginning with analysis of the IpaB/IpgC complex. Chemical crosslinking experiments demonstrated that the two IpaB *N*-terminal fragments and IpgC exist as homodimers when alone in solution, however, each IpaB fragment transitioned into heterodimeric complexes when combined with IpgC [6]. The addition of a *N*- or *C*-terminal cysteine to the IpaB fragments and subsequent sufhydryl-reactive labeling chemistry allowed the use fluorescence polarization measurements to characterize and compare the binding properties for the IpaB/IpgC complex. Interestingly, the binding between IpaB^1–226^ and IpgC was much stronger than for IpaB^28–226^/IpgC [6], supporting previous photocrosslinking experiments suggesting that IpaB contains multiple chaperone binding domains (CBDs) near the *N*-terminus [55]. Isothermal titration calorimetry confirmed these results and determined that the reactions were not only spontaneous, but endothermic and entropically driven. Because a co-crystal structure has remained elusive for this pair, FRET again provided a sensitive tool for probing IpgC binding effects on IpaB.

The single native tryptophan in IpaB (W105) provides a useful donor for intramolecular FRET to a sulfhydryl-reactive Alexa350 fluorophore bound to the engineered *N*- or *C*-terminal cysteine of each of the IpaB fragments shown to bind to IpgC. Compared to most extrinsic fluorophores, tryptophan has a relatively low quantum yield resulting in a *R*_o_ of only 21 Å for the tryptophan/Alexa350 FRET pair. However, with the small physical size of IpgC and the inherent 100% donor labeling efficiency, the use of tryptophan as a FRET donor was an ideal choice. As with any extrinsic FRET pair, efficiencies and corresponding distances were calculated by comparing donor (tryptophan) fluorescence intensity in the presence and absence of acceptor. FRET efficiencies were measured for each IpaB fragment alone as well as complexed with IpgC and are listed in Table 1 with corresponding intramolecular distances and resulting changes in distance following IpgC binding. The measured FRET efficiency and calculated distance between donor and acceptor for each isolated IpaB fragment was similar and very little change in donor-acceptor distance was seen when IpaB^28–226^ was incubated with IpgC to allow binding [6]. In contrast, the intramolecular distance between the FRET pair in IpaB^1–226^ increased approximately 10 Å once bound to IpgC. Taken together, these data define multiple chaperone binding regions with the *N*-terminal domain of IpaB. This highlights the importance of the extreme *N*-terminus in chaperone binding, and suggests that significant conformational changes occur in this region following IpgC interaction. Perhaps this elongation of the *N*-terminal residues is either directly responsible for reducing the ability for this region of IpaB to promote protein-protein interactions or is only a small part of a global conformational change that reduces the propensity of the protein to interact with other proteins prematurely. Several studies are currently underway to investigate these hypotheses and to better understand the multi-faceted roles of IpaB in the T3SS.

#### 4.3.4. Ongoing Studies Involving FRET Analysis of the *Shigella* T3SA Needle Tip

Because IpaB is ultimately delivered to the *Shigella* T3SA needle tip in the absence of IpgC, current studies are targeting interactions between IpaB and IpaD. These studies target not only the ability for these proteins to interact, but also the impact that this interaction has on the structure of each protein. This IpaB-IpaD interaction occurs at an exposed site at the T3SA needle tip which ultimately places IpaB in a position where it can sense contact with host cell membranes. We have shown that liposomes rich in cholesterol and sphingomyelin induce recruitment of the second translocator protein (IpaC) to the needle tip and results in full induction of secretion [33]. We have proposed that it is this exposed and oligomeric form of IpaB that is responsible for insertion into host cell membranes while remaining at the needle tip which allows it to become an integral part of the translocon pore that provides the conduit between the pathogen and host cell cytoplasm. Ongoing studies are aimed at characterizing the interaction between IpaB and phospholipid bilayers using a variety of fluorescence analyses. For example, FRET between a fluorescein donor covalently bound to full length IpaB and defined liposomes containing fluorescent phospholipid acceptors is providing information on the requirements for IpaB interaction with membranes and pore formation within these membranes. These studies have proven useful in complementing a series of other biophysical techniques such as liposome flotation and disruption assays to identify and characterize the interaction between defined lipid membranes and various oligomeric states of IpaB. While such studies are in their early stages, FRET is continuing to be an instrumental tool in understanding the events that occur within the *Shigella* T3SA needle tip and how they affect host interactions.

### 4.4. The Future of FRET in Bacterial Pathogenesis

**Figure 6:**
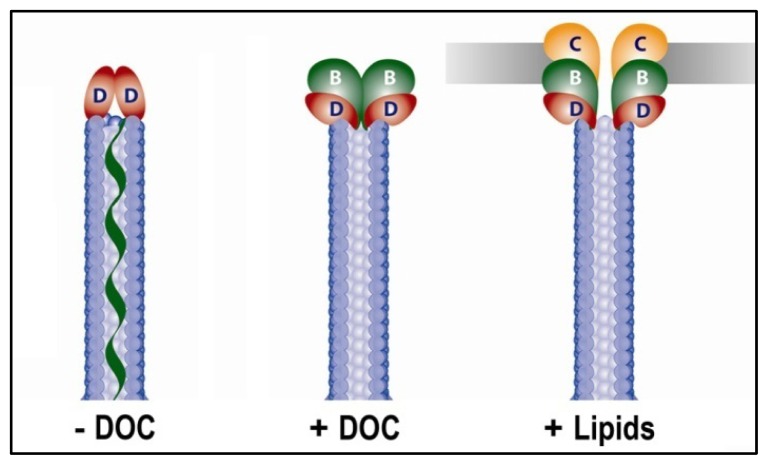
Schematic of the *Shigella flexneri* T3SA needle and tip complex illustrating the proposed stepwise mechanism for maturation. While the mechanism of terminating needle assembly remains unclear, it is known that a pentamer of IpaD constitutively resides at the tip of the T3SA following termination. IpaD acts as an environmental small molecule sensor that specifically binds the bile salt deoxycholate (DOC), resulting in conformational changes in IpaD that signal the recruitment of IpaB which is residing within the MxiH needle channel as well as residing as a heterodimeric complex with the chaperone IpgC in the bacterial cytoplasm. Recruitment of IpaB then leads to the second stable form of the tip complex where it awaits contact with a host cell plasma membrane and/or membrane components such as cholesterol, recruiting the second hydrophobic translocator IpaC to the tip complex. Together, IpaB and IpaC complete the translocon pore that spans the host membrane, completing the unidirectional conduit connecting the *Shigella* cytoplasm to the host cell cytoplasm. This event occurs either concomitantly with or directly preceding the full onset of secretion of T3SS effectors into the host cell. Figure adapted from [2].

The T3SS tip complex represents a challenging, but important target of study in bacterial pathogenesis. The structural conservation of T3SA across broad species boundaries ranging from *Chlamydiae*[56,57] to the more genetically complex *Burkhoderia* spp [58]. to the Gram-negative bacterial flagellar systems [59] has provided a strong argument for the evolutionary importance of the T3SA and this secretion system in general. An advantage afforded to *Shigella* over these other systems for studying the steps of type III secretion induction include existence of a single T3SS (and no flagella) and the identification of extracellular stimuli that give rise to the multiple static steps of secretion induction. Another advantage for our group has been the ability to explore the individual components of the mature T3SA tip complex *in vitro* to solve many aspects of their structures, interactions, and the influence that the binding of ligands and protein partners has on their conformational state. FRET has been a valuable tool, among many, that has provided us with a unique perspective of the molecular events that occur prior to type III secretion induction in *Shigella*. Here, we have discussed a series of experiments specifically utilizing FRET to dissect the mechanism of the T3SA tip complex maturation in *Shigella* which is broken down into a three step working model in Figure 6.

A future challenge is to take what we know of the *Shigella* system and extend it across species boundaries. Together with more traditional biochemical techniques, many innovative studies have proven fruitful in understanding this complex process in multiple systems. For example, the Enninga lab has recently developed a sensitive â-lactamase cleavable FRET reporter system (CCF4) capable of monitoring pathogen exposure to host cell cytoplasm. The CCF4 reporter passively diffuses across the host membrane where cytoplasmic esterases convert it to an anionic form of the reporter that remains within the cytoplasm. Exposure of the reporter to â-lactamase on the surface of the studied bacterium results in cleavage of the reporter, physically separating the FRET pair engineered at either end of the reporter, thus relieving energy transfer and donor quenching. This increase in donor fluorescence has been measured with both high throughput flow cytometry and real-time fluorescence microscopy techniques to follow T3SS dependent host cell vacuolar escape by *Shigella flexneri*[60] and lysosomal rupture by *Mycobacterium tuberculosis*[61], respectively. Studies such as these illustrate the continuing utility of FRET when used creatively to address traditionally difficult questions.

## 5. Conclusions

FRET has provided a powerful tool for studying biological systems including bacterial pathogenesis at a molecular level. This review focuses specifically on the use of FRET in studying the stepwise maturation of the *Shigella* T3SA tip complex. The small dimensions, protein dynamics, and sequentially changing composition of the T3SA make it a challenging system to study even with many of the advanced technologies currently available, however, FRET has proven invaluable in providing the sensitivity and flexibility needed for dissecting the molecular interactions and protein conformational changes involved in T3SA maturation. In reviewing these specific FRET-based studies, it is clear that such experiments require a high level of planning and the results warrant careful interpretation. As with the use of any sophisticated biophysical method, proper controls and complementary supporting experiments are needed to ensure confidence in the conclusions drawn based on FRET analyses. Through the development of ever-improving fluorophores, instrumentation, and creativity, the use of FRET is well suited to provide a detailed understanding of the interactions and dynamics that occur within the macromolecular complexes involved in bacterial pathogenesis. It is our goal in this review to provide a starting point from which other investigators may use FRET for better understanding the mechanisms that govern bacterial pathogenesis.

## Figures and Tables

**Table 1:** Using FRET to determine changes in intra distances within IpaB.

Protein	No IpgC		1 ìM IpgC		Är (Å)
	
	FRET a (%)	r (Å)	FRET (%)	r (Å)	
IpaB^N28.226^	47.1 ± 3.2	21.4	37.9 ± 14.8	22.8	1.4
IpaB^C28.226^	74.5 ± 5.9	17.6	46.1 ± 6.8	21.6	4.0
IpaB^N1.226^	37.6 ± 3.3	22.8	6.5 ± 3.6	32.8	10.0

a FRET efficiency was measured using the intrinsic probe W105 as the donor for IpaB^28.226^ and IpaB^1.226^ to either *N*- or *C*-terminal Alexa350 acceptor probes covalently linked to an engineered terminal Cys residue [6]. FRET efficiencies were calculated using Equation (4) and calculated distances (*r*) were determined according to Equations (2). Values compiled from [6].
